# The Evolving Landscape of GEP-NENs in the Era of Precision Oncology: Molecular Insights into Tumor Heterogeneity

**DOI:** 10.3390/cancers17132080

**Published:** 2025-06-21

**Authors:** Sunanda Biswas Mukherjee, Rachyl M. Shanker, James P. Madigan, Samira M. Sadowski

**Affiliations:** Neuroendocrine Cancer Therapy Section, Surgical Oncology Program, Center for Cancer Research, National Cancer Institute, National Institutes of Health, Bethesda, MD 20892, USA; sunanda.mukherjee@nih.gov (S.B.M.); rachyl.shanker@nih.gov (R.M.S.); james.madigan@nih.gov (J.P.M.)

**Keywords:** gastroenteropancreatic neuroendocrine neoplasms (GEP-NENs), tumor heterogeneity, metastasis, multi-omics, artificial intelligence (AI), precision oncology

## Abstract

Gastroenteropancreatic neuroendocrine neoplasms (GEP-NENs) are a varied group of tumors that originate from neuroendocrine cells found throughout the gastrointestinal tract. These tumors encompass a broad spectrum of biological behaviors, ranging from slow-growing, well-differentiated neuroendocrine tumors (GEP-NETs) to aggressive and poorly differentiated neuroendocrine carcinomas (GEP-NECs), complicating their accurate classification and effective treatment. While advances in molecular research have refined our understanding of these tumors, their complexity, unpredictable progression, and differential response to therapies remain major clinical hurdles. A significant clinical challenge is the accurate grading and diagnosis of GEP-NENs, which is traditionally reliant on subjective methods. However, innovative technologies, such as artificial intelligence-based diagnostics, multi-omics approaches, and precision oncology, are now offering solutions for more precise and reliable classification. Meanwhile, emerging therapies aiming to activate the immune response or modify the tumor environment present promising avenues for improved outcomes. Realizing the full potential of these advances will require a thoughtful integration of molecular insights with standardized diagnostic practices and evolving therapeutic strategies, ensuring that progress in research meaningfully informs and enhances patient care across diverse clinical settings. This review discusses new advancements and explores future directions toward personalized and effective treatments for GEP-NENs.

## Simple Summary

Gastroenteropancreatic neuroendocrine neoplasms (GEP-NENs) arise from neuroendocrine cells in the digestive system and show a wide range of growth patterns. While traditional methods continue to guide diagnosis and grading, they often fall short of capturing the full biological complexity of these tumors. In this review, we explore how advances in multi-omics technologies, artificial intelligence, and precision oncology are opening new doors to more accurate classification, a deeper understanding of tumor heterogeneity, and the development of novel therapeutic strategies. By connecting emerging science with clinical practice, we aim to highlight a future where patients with GEP-NENs can benefit from truly personalized care.

## 1. Introduction

Gastroenteropancreatic neuroendocrine neoplasms (GEP-NENs) are a diverse group of rare tumors arising from specialized neuroendocrine cells within the pancreas and the gastrointestinal (GI) tract [1,2]. These tumors encompass two primary categories with distinct clinical behaviors: well-differentiated neuroendocrine tumors (GEP-NETs), characterized by slower growth and less aggressive features, and poorly differentiated neuroendocrine carcinomas (GEP-NECs), which exhibit rapid progression, aggressive invasion, and resistance to standard therapies [3]. Clinically, the heterogeneity of GEP-NENs presents substantial challenges in accurate classification, prognosis, and treatment.

Surgery is the only potential curative treatment for GEP-NENs, particularly for locoregional tumors, aiming for complete resection. In cases of limited metastatic disease with resectable primary tumors, the surgical removal of both primary and metastatic lesions is considered. For small or peripherally located tumors, less extensive surgery like enucleation or endoscopic resection may be appropriate [4,5]. GEP-NETs frequently express somatostatin receptors (SSTRs), rendering them responsive to targeted therapies such as somatostatin analogs and peptide receptor radionuclide therapy (PRRT) like ^177^Lu-DOTATATE [6,7,8,9]. Conversely, GEP-NECs rarely express these receptors, necessitating more intensive systemic approaches like platinum-based chemotherapy. Targeted agents such as everolimus and sunitinib have become standard for progressive NETs, and capecitabine-temozolomide (CAPTEM) is increasingly used in pancreatic NETs. Additionally, locoregional approaches like trans-arterial chemoembolization (TACE) and trans-arterial embolization (TAE) provide targeted tumor control and symptom relief in liver-dominant disease [10,11].

**Figure 1:**
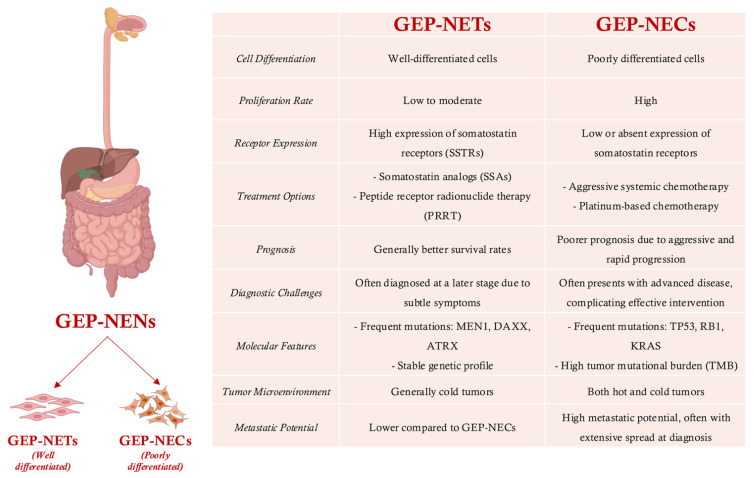
Overview of key molecular differences in GEP-NETs and GEP-NECs.

The stark differences in receptor expression and treatment responsiveness between GEP-NETs and GEP-NECs highlight the necessity of precise tumor classification to inform optimal therapeutic decision-making (Figure 1). The diagnosis and grading of GEP-NENs are often complicated by overlapping clinical presentations, late-stage detection, and variability in tumor behavior [12]. Although histological grading based on proliferation indices such as Ki-67 is standard, inconsistencies in scoring frequently lead to misclassification, impacting patient management decisions [13,14]. Furthermore, survival rates for GEP-NEN patients vary considerably based on tumor grade, location, and metastatic status, underscoring the critical need for early and accurate staging to guide effective treatment strategies and targeted therapy [15,16].

Recent advances in molecular and genomic research have provided deeper insights into the mechanisms driving tumor progression and therapy response. Genetic alterations, including mutations in key tumor suppressors and oncogenes, epigenetic modifications, and significant shifts in tumor immune microenvironment dynamics, have emerged as key factors influencing tumor behavior and treatment response. These molecular insights not only enhance our understanding of GEP-NEN tumor heterogeneity but also open new avenues for personalized treatments. In parallel, artificial intelligence (AI), machine learning (ML), and digital pathology are transforming diagnostic precision and enabling the high-throughput, reproducible analysis of histological and molecular data.

Given the rapid evolution of both diagnostic and therapeutic tools in GEP-NENs, we believe that the most immediate clinical priority lies in improving diagnostic accuracy and molecular classification, as these are fundamental to guiding treatment. While emerging technologies like AI and digital pathology hold promise, their adoption should be gradual based on clinical utility, feasibility, and validation within real-world settings. This review bridges established clinical knowledge with advances in AI and omics technologies, offering a practical yet progressive framework that highlights innovative diagnostic and therapeutic strategies to improve GEP-NEN management.

## 2. Advancing Grading and Classification of GEP-NENs: Molecular Insights, Challenges, and Technological Innovations

Understanding the grading systems for GEP-NENs is crucial for navigating their complex classification, which is primarily based on the degree of differentiation [2]. Well-differentiated neuroendocrine tumors are categorized into three distinct grades based on their proliferation rates: Grade 1 (G1) tumors have low proliferation rates, with a Ki-67 labeling index below 2%. Moving up in severity, Grade 2 (G2) tumors show a moderate proliferation rate, with a Ki-67 index between 3% and 20%. The most aggressive of these, Grade 3 (G3) tumors, present with a high proliferation index exceeding 20%, signaling a significant challenge in management and prognosis [2]. Poorly differentiated neuroendocrine carcinomas are highly aggressive, exhibit significant nuclear atypia and mitotic activity, and are further subclassified into small-cell and large-cell types. Another distinct category, mixed neuroendocrine non-neuroendocrine neoplasms (MiNENs), contains both neuroendocrine and non-neuroendocrine components, often with an adenocarcinoma component [17,18,19]. This dual presence introduces additional complexity into their diagnosis and management. Diagnosing MiNENs poses significant challenges, particularly when relying on limited biopsies or cytological samples that may not capture both tumor components adequately. Cytology samples typically provide insufficient cellularity and architectural context necessary for distinguishing the two distinct components clearly. Furthermore, MiNENs often exhibit substantial intratumoral heterogeneity, meaning that small biopsy samples may capture only a single component, leading to diagnostic misclassification. Immunohistochemical profiling can aid in the detection of neuroendocrine differentiation, but it is limited by scant cellular material, often failing to definitively classify the lesion. Therefore, the accurate diagnosis of MiNENs frequently necessitates correlation with surgical resection specimens, comprehensive immunohistochemistry, and sometimes multiregional biopsies to ensure the proper representation of both tumor components [20,21].

These classification systems have been shaped by significant contributions from global health organizations. Earlier versions, like the World Health Organization (WHO) system, categorized tumors based on clinical behavior, which proved effective for resected specimens but limited for advanced cases [22,23,24,25,26,27,28,29]. Subsequent updates by the European Neuroendocrine Tumor Society (ENETS) and the North American Neuroendocrine Tumor Society (NANETS) incorporated a Tumor-Node-Metastasis (TNM) staging system, mitotic count, and the Ki-67 index, allowing for a better evaluation of tumor aggressiveness, even from small biopsies [30,31,32]. The WHO’s 2010 revision formally distinguished NETs from NECs and adopted a three-tier grading scale [26,33,34]. The most recent 2022 WHO classification added histological and molecular parameters to improve diagnostic clarity [35]. However, several practical challenges persist. The Ki-67 index, although widely used, is prone to variability due to inter-observer differences, sampling bias, and the manual selection of “hotspot” regions [36,37,38,39]. These issues are particularly problematic when distinguishing between G1 and G2 tumors, where small differences in Ki-67 can influence treatment strategies. Such variability underscores the need for more objective and reproducible grading approaches. Furthermore, WHO’s recent recognition of well-differentiated Grade 3 NET as distinct from poorly differentiated NECs has introduced additional complexity. These two tumor classifications often exhibit overlapping molecular features, such as TP53 mutations or the loss of Rb1 expression, and similarly elevated Ki-67 levels. This overlap complicates accurate diagnosis, especially when tissue availability is limited, such as in small biopsies or metastatic samples. While histological assessment combined with Ki-67 and immunohistochemical markers (p53, Rb1) is the current recommendation, diagnostic ambiguity remains common, highlighting the continued need for more robust, objective, and reproducible grading methods [40].

GEP-NECs are categorized distinctly within the TNM, similar to other NENs of the GI tract, yet their classification draws a closer parallel to small-cell lung cancer (SCLC) and other aggressive extrapulmonary neuroendocrine carcinomas, due to their aggressive nature [41,42]. Unlike GEP-NETs, where tumor size and differentiation play a major role in staging, the staging of GEP-NECs is primarily centered on how extensively the cancer has spread throughout the body. In cases of localized disease, the tumor remains confined to the original site and, if detected early, may still be eligible for surgical removal [43]. However, due to the rapid growth and highly invasive nature of these tumors, achieving complete surgical resection is often challenging. Even when the tumor appears localized, microscopic metastases may already be present, making early intervention critical [44]. On the other hand, extensive disease refers to cases where the cancer has metastasized widely, to distant organs such as the liver, lungs, or bones, making surgical removal impractical [45]. At this stage, treatment is largely palliative, focusing on slowing disease progression and managing symptoms, rather than achieving a cure and are considered as high-grade malignancies [1]. Their rapid growth and poor differentiation distinguish them from well-differentiated NETs, making them particularly aggressive [46,47].

GEP-NENs display remarkable variability in outcomes. Patients with localized, well-differentiated Grade 1 tumors often achieve 10-year overall survival (OS) rates of 80–90%, particularly when arising in the appendix or rectum [48]. By contrast, once metastases develops or when tumors are poorly differentiated (Grade 3 NECs), the 5-year OS plunges below 10%, underscoring the aggressive nature of high-grade disease [48]. Younger patients (<50 years) tend to live longer across all grades and sites, with many early-onset rectal cases still alive at the last follow-up, whereas pancreatic early-onset tumors show a median OS around 11 years [49]. Beyond age and stage, important predictors include the Ki-67 proliferation index; primary tumor location, the best in appendiceal/rectal regions and the worst in pancreatic regions; and molecular features such as TP53 mutations or Rb1 loss, which drive poorer outcomes [48,49,50]. The retention of somatostatin receptor expression also portends longer survival by enabling effective targeted therapies [51].

Emerging technologies like artificial intelligence, digital pathology, and multi-omics are now offering promising solutions to these grading and classification challenges. Advances in digital pathology have led to the development of machine learning tools that can dynamically identify and quantify Ki-67 hotspots, addressing the variability introduced by manual field selection. Tools such as QuPath [52] and HALO [53] apply pixel-based learning and convolutional neural networks to automate hotspot detection based on localized proliferative activity. This allows for a more consistent and reproducible assessment, enabling real-time automated Ki-67 quantification, reducing human error, and enhancing reproducibility [54,55,56]. When paired with omics data, like genomics, transcriptomics, and proteomics, these platforms could provide a deeper molecular understanding of tumor biology, improving subtype differentiation between NETs and NECs [57,58,59]. Importantly, the whole-transcriptome sequencing of routinely archived FFPE blocks has already proven feasible in other malignancies. For example, the bulk RNA-seq of 90 FFPE osteosarcoma specimens robustly identified 530 shared differentially expressed genes versus matched normal tissue, demonstrating that formalin-fixed material yields high-quality transcriptomes suitable for retrospective analyses [60]. Another study demonstrated that whole-transcriptome sequencing can be robustly applied to archival FFPE adrenocortical tumor specimens, uncovering distinct gene expression signatures that correlate with tumor subtype and clinical outcomes [61].

**Figure 2:**
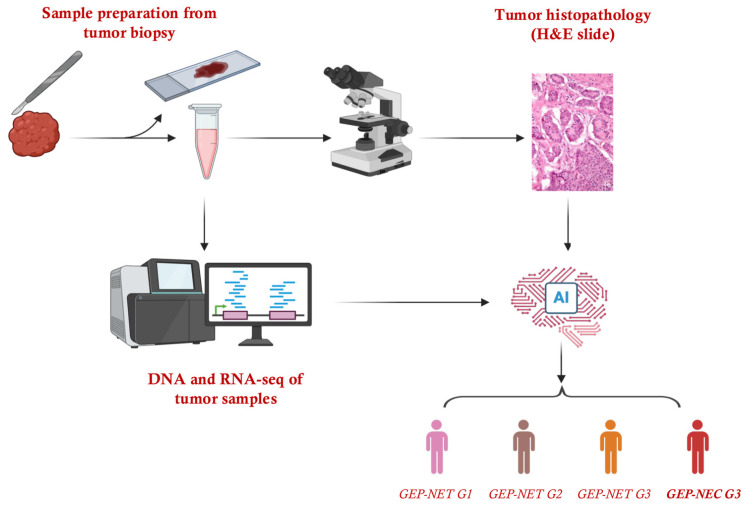
Integrating omics, digital pathology, and AI for improved grading and classification of GEP-NENs.

Further, AI-based systems also facilitate the longitudinal tracking of tumor changes, offering insights into tumor progression, dedifferentiation, or treatment response over time [62]. Another emerging technology is integrating imaging data with other omics information, which is used for the extraction of subtle imaging features using computational algorithms, representing another promising tool for improving the classification and prognosis of GEP-NENs. Radiomic analysis can objectively capture patterns within CT, MRI, or PET scans that are not visible to the human eye, providing additional layers of biological insight beyond standard visual assessments [63]. When combined with AI, radiomics may enhance current classification schemes by correlating imaging phenotypes directly with molecular characteristics and clinical outcomes [64]. Early studies suggest that radiomic signatures might help differentiate aggressive neuroendocrine carcinomas from less aggressive well-differentiated tumors, facilitating personalized treatment decisions [63]. Despite its promise, the practical integration of radiomics into clinical routines will require standardized imaging protocols, robust validation across institutions, and algorithms tailored specifically to neuroendocrine tumor biology. While not yet standard in clinical practice, integrating these advanced technologies represents a major step toward a more personalized, precise, and consistent management of GEP-NENs. By incorporating these integrated approaches, we can advocate for a shift towards a more data-driven, patient-specific strategy in managing these complex cancers, potentially setting a new standard that promises improved outcomes and better-informed therapeutic decisions (Figure 2).

## 3. Tumor Heterogeneity and Therapy Response in GEP-NENs: Challenges and Emerging Solutions

Tumor heterogeneity in GEP-NENs presents significant challenges in management due to genetic, cellular, and microenvironmental diversity that affects tumor behavior, progression, and responses to treatment [65,66]. Variability, not only between primary and metastatic tumors, but also within tumors themselves, often leads to inconsistencies in standard grading practices like those based on Ki-67 markers, complicating accurate diagnosis and effective treatment planning [67,68]. As a result, clinicians often struggle to predict how a given tumor will behave and respond to therapy, underscoring the need for more personalized management strategies.

Current treatment decisions for GEP-NETs are largely determined by tumor grade, location, and stage, with surgical resection being the preferred approach for localized disease [12,69]. However, systemic therapies are required for metastatic conditions, including somatostatin analogs, such as octreotide and lanreotide, which target somatostatin receptors, to mitigate hormone-related symptoms and slow tumor growth [70]. SSAs therapeutically function by reducing hormone-related symptoms and slowing tumor growth, as demonstrated in key trials, such as PROMID [71] and CLARINET [72]. Despite their efficacy, resistance to SSAs is common, potentially due to epigenetic changes affecting receptor expression, prompting research into combinations with epigenetic-modifying drugs to overcome this challenge. Additionally, peptide receptor radionuclide therapy, with agents like ^177^Lu-DOTATATE, serves as an effective second-line treatment when disease progresses or SSAs fail [73]. Ongoing investigations aim to enhance these therapeutic options by targeting new somatostatin receptor subtypes and understanding resistance mechanisms, striving to improve outcomes for patients with this complex disease [74,75]. Resistance to these therapies is often driven by adaptive epigenetic reprogramming that downregulates receptor expression or activates alternative survival pathways. Ongoing efforts using multi-omics approaches aim to unravel these mechanisms in real time, with the goal of guiding combination strategies that preempt or overcome therapeutic escape.

One promising strategy to surmount SSA resistance is to pair SSAs with epigenetic-modifying drugs, such as histone deacetylase (HDAC) inhibitors or DNA methyltransferase (DNMT) inhibitors. The rationale is that epigenetic reprogramming might restore somatostatin receptor expression and resensitize tumors to SSAs and PRRT. Early studies of this combination approach have shown some tumor redifferentiation and improved receptor targeting, but toxicity has emerged as a key challenge [75,76,77]. Parallel efforts are focused on discovering new somatostatin receptor targets and further elucidating resistance mechanisms, given their importance for improving patient outcomes. Notably, recent findings demonstrate that somatostatin receptor expression can indeed be epigenetically modulated. In small intestinal neuroendocrine tumors (SI-NETs), the SSTR2 gene promoter is significantly hypomethylated compared to normal tissue, accompanied by reduced levels of the repressive histone mark H3K27me3 changes that correlate with higher SSTR2 mRNA expression [78]. In contrast, the activating mark H3K9ac remains unchanged, suggesting a specific loss of repressive chromatin at the SSTR2 locus. Complementing these findings, the in vitro treatment of NET cell lines with histone deacetylase inhibitors such as valproic acid robustly upregulates SSTR2 surface levels, confirming that chromatin remodeling can sensitize tumors to PRRT [9].

For patients with advanced GEP-NETs, the treatment landscape has significantly evolved, integrating targeted therapies such as mTOR inhibitors, such as everolimus, and tyrosine kinase inhibitors (TKIs), such as sunitinib, which have shown effectiveness in blocking key molecular pathways that drive tumor growth, as evidenced by clinical trials, such as the RADIANT studies [79]. Despite their success, a major hurdle remains in the form of drug resistance, with tumors often activating alternative survival pathways or adapting mechanisms, like autophagy to circumvent these treatments [80,81,82,83]. Autophagy has been shown to support tumor survival in GEP-NENs under mTOR inhibition. In BON-1 pancreatic NET cells, everolimus increased autophagic markers such as LC3-II and autophagic vacuoles, while autophagy inhibition via chloroquine or ATG5/7 knockdown enhanced apoptosis [84]. Similar findings were observed in patient-derived pancreatic NET organoids, where everolimus induced p62 degradation and elevated autophagic flux, highlighting autophagy as a key resistance mechanism [85]. To address potential resistance issues, researchers are exploring combined treatment strategies that include PRRT with radiosensitizers, epigenetic modifiers [86], or next-generation therapies, such as alpha-emitting radionuclides and somatostatin antagonists. In particular, alpha emitters demonstrate superior cytotoxicity and can overcome resistance to beta-based PRRT. In early trials, ^213^Bi-DOTATOC induced responses in patient’s refractory to ^177^Lu-DOTATOC, with manageable toxicity [87]. Long-term outcomes with ^225^Ac-DOTATOC showed durable control and tolerable side effects, even in heavily pre-treated patients [88]. Preclinical models further suggest that alpha emitters inflict more lethal DNA damage than beta emitters, and their anticancer activity can be synergistically enhanced by combining them with PARP inhibitors [89].

Despite these promising developments in radionuclide therapy, a subset of patients, particularly those with high-grade or poorly differentiated tumors, continue to exhibit limited responses, underscoring the need for alternative systemic strategies. In this context, immune checkpoint inhibitors (ICIs) have emerged as an area of active investigation. While single-agent ICIs have shown limited efficacy in GEP-NENs, particularly in well-differentiated tumors, combination regimens such as ipilimumab plus nivolumab have demonstrated modest clinical benefit in high-grade GEP-NECs, with an objective response rate of 14.7% and disease control observed in over 40% of patients [90]. These findings suggest that dual ICB may hold potential for select patients, especially those with more immunogenic tumor profiles.

Alongside immunotherapy, chemotherapy regimens such as CAPTEM remain a cornerstone in the treatment of metastatic or progressive NETs. CAPTEM combinations continue to be evaluated in clinical trials such as ECOG-ACRIN EA2142, reflecting a growing interest in optimizing systemic treatment strategies for advanced disease. In parallel, locoregional therapies, including TAE and TACE remain essential options, particularly in patients with liver-dominant tumors, where they can provide meaningful symptom relief and disease control [91,92,93,94,95,96,97]. One study with 148 patients found that selective internal radiation therapy (SIRT) led to a positive response in 62.9% of cases, with stable disease in 22.7% [98]. Although the treatment options for GEP-NETs are still somewhat limited, there is a lot of ongoing research aimed at finding novel therapies, exploring innovative treatment combinations, and evaluating how well existing treatments work. Clinical trials are focused on tackling the challenges brought about by the complexity and varied nature of these tumors, with the ultimate goal of improving treatment strategies and boosting patient outcomes [99,100]. Notably, the NETTER-2 trial is currently evaluating the combination of PRRT (^177^Lu-DOTATATE) with octreotide versus octreotide alone in advanced GEP-NETs, potentially reshaping second-line treatment options [101]. Furthermore, immunotherapy studies, including combinations of immune checkpoint inhibitors such as ipilimumab and nivolumab, are being tested specifically for high-grade NECs, aiming to leverage their distinct immune profiles to improve therapeutic outcomes [55,56].

Tumor heterogeneity in GEP-NECs contributes significantly to treatment resistance, driven by genetic mutations, phenotypic plasticity, and the evolving tumor microenvironment (TME). Key genetic alterations, including *TP53* (64%), *RB1* (14%), *KRAS* (22%), *BRAF* (63% in colorectal NECs), and *MYC* amplifications (51%), fuel aggressive tumor behavior and resistance to therapy [51,77,102]. Phenotypic plasticity, where tumors dynamically shift between well and poorly differentiated states, further complicates treatment, particularly under therapeutic pressure [103,104,105]. Within the TME, immune evasion mechanisms, such as reduced MHC-II expression in nonpancreatic NECs and hypoxia-driven metabolic adaptations, create additional barriers to treatment success [106,107]. Clonal evolution under chemotherapy, particularly in high Ki-67 tumors, leads to the emergence of resistant subclones, undermining the long-term efficacy of platinum-based regimens. With limited modest success, ICIs remain largely ineffective in GEP-NECs, due to low neoantigen diversity and immune heterogeneity, necessitating biomarker-driven patient selection [83,84]. Emerging strategies, including PARP inhibitors, targeting DNA damage response, and EZH2 inhibitors, to modulate epigenetic plasticity, offer new avenues to tackle therapy resistance [102,108]. However, critical gaps remain, particularly in defining second-line treatments for platinum-refractory cases and understanding epigenetic regulation in tumor adaptation. Moving forward, integrating molecular profiling, immune TME characterization, and novel epigenetic-targeting strategies into clinical practice will be essential for developing precision medicine approaches tailored to GEP-NEC patients.

To meet the need for more precise therapeutic tailoring, state-of-the-art molecular profiling tools are being deployed to dissect GEP-NEN heterogeneity at high resolution. In particular, single-cell RNA sequencing (scRNA-seq) enables the analysis of thousands of individual tumor cells, thereby revealing rare subpopulations including those that may drive relapse or drug resistance and mapping clonal evolution under treatment pressure [109,110]. This approach has already provided important insights. For example, scRNA-seq of 24,544 cells from a Grade 2 PNET delineated multiple cell populations within the tumor, malignant cells along with diverse immune and stromal components, illuminated hypoxia-driven progression pathways, and identified a gene expression signature involving *PCSK1* and *SMOC1* that predicted metastatic potential [111]. Another scRNA-seq study on SI-NETs and a mixed lung neuroendocrine tumor identified distinct epithelial-like and neuronal-like subtypes, uncovered proliferative immune cells, and revealed progenitor populations co-expressing neuroendocrine and squamous markers [112]. These high-resolution genomic profiles underscore the profound cellular diversity in GEP-NENs and provide clues as to how specific subclones contribute to disease progression.

When multi-region sequencing uncovers distinct clonal populations within a single tumor, treatment should initially target early driver mutations shared across all samples since it represents the tumor’s core biology. At the same time the serial monitoring of circulating tumor DNA can reveal emerging subclones in real time, allowing clinicians to adjust therapy by adding or switching to agents that inhibit newly dominant pathways. Incorporating time-series or multi-region biopsies can further enhance decision-making by revealing how clonal and epigenetic shifts evolve under treatment pressure [113,114,115]. More recently, spatial transcriptomics and multiplexed imaging techniques have emerged as transformative tools, allowing for the simultaneous assessment of gene expression and cellular architecture within the intact tumor microenvironment [116]. Recent advances in spatial profiling have made it possible to better understand how tumor cells interact with their surrounding environment in GEP-NETs. A study using NanoString’s Digital Spatial Profiling on standard FFPE tissue samples found that pancreatic NETs tend to be more immune-infiltrated, with higher levels of CD3^+^ T cells and CD68^+^ macrophages, while duodenal gastrinomas appeared largely immune-cold. Interestingly, even in the absence of immune cells, gastrinoma tumor cells were found to produce IL-17B, which can activate inflammatory pathways like NF-κB/STAT3. These results not only validate spatial profiling on routine clinical blocks but also reveal how GEP-NETs establish distinct neuro-immune microenvironments that may influence progression and therapeutic response [117]. By integrating single-cell and spatial multi-omics data, researchers can now construct a comprehensive map of tumor evolution, immune dynamics, and microenvironmental influences. This integrative strategy holds immense promise for identifying predictive biomarkers, understanding diverse treatment responses, and discovering novel therapeutic targets tailored to specific subtypes and disease stages, ultimately advancing precision medicine for GEP-NENs.

## 4. Metastasis in GEP-NENs: Molecular Heterogeneity Drives Divergent Tumor Trajectories

GEP-NENs exhibit significant molecular heterogeneity that underlies their diverse metastatic behaviors [50,103]. Tumor metastasis is a complex, multi-stage process involving initial tumor cell invasion, entry into circulation, survival within the bloodstream, and eventual colonization at distant sites [118]. Among GEP-NEN subtypes, GEP-NECs are characterized by their aggressive nature and rapid progression, largely due to enhanced epithelial-to-mesenchymal transition (EMT) processes that facilitate their invasion and spread [119,120]. The aggressive metastatic behavior in GEP-NECs is further driven by robust angiogenesis, where the rapid development and expansion of new blood vessels are critical, due to their high growth demands. This heightened angiogenic activity is primarily mediated by factors such as vascular endothelial growth factor (VEGF) and endoglins, which not only promotes vascularization but also aids in the tumor cells’ dissemination into the systemic circulation [120]. Molecular signaling pathways, like PI3K/Akt/mTOR, are particularly active in GEP-NECs, contributing significantly to their metastatic potential and the development of resistance against targeted therapies [10]. These pathways, often intensified by mutations in genes like *TP53*, *RB1*, and *MYC*, underline the challenges in treating GEP-NECs, as they adapt quickly to therapeutic pressures, necessitating advanced strategies like combination therapies, targeting multiple aspects of tumor growth and survival [121]. While GEP-NETs also exhibit molecular heterogeneity, they generally have a more variable range of metastatic behaviors and a slightly less aggressive course. The metastatic process in GEP-NETs involves not only EMT, where the epithelial tumor cells adopt a mesenchymal phenotype, enhancing their invasive potential and facilitating tissue infiltration, but also the reverse transition mesenchymal-to-epithelial transition (MET) at distant sites, which is critical for the establishment of secondary tumors [122,123]. Key regulators of EMT, such as Snail, Slug, and Twist, repress epithelial markers (e.g., E-cadherin) and promote mesenchymal markers, contributing directly to tumor cell motility and invasiveness [124,125]. Similarly to GEP-NECs, angiogenesis plays a significant role in GEP-NET metastasis, supported by angiogenic factors such as VEGF, fibroblast growth factor (FGF), and platelet-derived growth factor (PDGF) [69,119,121,126]. These angiogenic factors not only promote tumor vascularization but also facilitate tumor cell dissemination into systemic circulation. Although VEGF expression correlates with increased tumor vascularization, recent evidence suggests that other angiogenic regulators, notably endoglin, may play even more significant roles in metastasis and tumor aggressiveness [120].

At the molecular signaling level, the PI3K/Akt/mTOR pathway plays a key role in driving metastasis across various GEP-NET subtypes, including pancreatic neuroendocrine tumors (pNETs). This pathway’s activation, frequently spurred by growth factors and compounded by mutations in genes like *PTEN*, *TSC2*, and *PIK3CA*, plays a crucial role in tumor proliferation, survival, and the metastatic process [127,128]. While genetic studies highlight the consistent presence of mutations in *MEN1*, *DAXX*, and *ATRX* in pNETs, which contribute to their chromatin remodeling and telomere maintenance, distinct patterns emerge in metastatic versus primary tumors. Metastatic pNETs often show increased mutations in *TP53*, *KRAS*, and *RB1*, indicating a shift toward more aggressive behavior. Further, genomic analysis reveals a significant allelic loss of chromosome 18q in midgut NETs, a factor linked to poor prognosis and advanced disease progression [129,130,131]. Additionally, transcriptomic analyses underscore that metastases display heightened activity in cell proliferation pathways, while primary tumors maintain characteristics conducive to metastatic potential, such as EMT and TGF-β signaling [51,132,133]. Metastatic SI-NETs are often driven by chromosomal instability, rather than high mutation rates, with the loss of chromosome 18q leading to reduced *SMAD4* expression and an increased risk of metastasis. While pNETs frequently show *CDKN2A* loss, SI-NETs are more commonly associated with *CDKN1B* mutations, though their direct impact on protein function is still unclear. Recurrent chromosomal alterations, including losses on chromosomes 18, 11, and 16 and gains on chromosomes 4, 5, 14, and 20, further highlight the genomic complexity of these SI-NETs tumors. Though mutations in *TP53*, *RB1*, and *KRAS* do occur in metastatic SI-NETs, they are less frequent compared to their metastatic pNET counterparts [134,135].

Collectively, these molecular insights underscore how genomic, transcriptomic, and pathway-level heterogeneity shapes metastatic potential and clinical outcomes across different GEP-NEN subtypes. Even with limited sample sizes, as in case of GEP-NENs, integrative genomic profiling remains a powerful tool to dissect tumor heterogeneity. Through bulk and single-cell RNA sequencing, it becomes feasible to stratify GEP-NENs into molecularly distinct subtypes, revealing variations in signaling pathways, receptor expression, and immune infiltration. This molecular layering helps refine prognosis and tailor therapies, even in rare or heterogeneous tumor populations. Recent studies have shown that even small cohorts, when analyzed using advanced genomic tools, can yield robust and clinically meaningful stratifications, particularly when integrated with imaging and pathology data [46,136,137,138].

Different subtypes, and even tumors from the same patient, can follow very different metastatic paths driven by unique genetic and molecular changes. This diversity helps explain why some tumors spread quickly, or resist treatment, while others do not. Understanding these differences at the molecular level is essential. Without a better understanding of the molecular drivers of tumor heterogeneity, we may risk missing key facilitators of disease progression and resistance. Moving forward, bringing this molecular understanding into clinical decision-making may help better predict how a tumor will behave, identify which patients are likely to respond to certain therapies, and ultimately guide more personalized and effective treatment strategies.

## 5. Integrating Molecular Insights into Clinical Practice for GEP-NENs: Bridging Diagnostic Gaps and Advancing Therapeutic Strategies

GEP-NENs represent a remarkably diverse group of tumors that differ not only in their anatomical location and histological grade, but also in their clinical behavior and therapeutic response. This heterogeneity often complicates timely diagnosis and effective disease management. Functional tumors such as insulinomas, gastrinomas, VIPomas, and carcinoid tumors can present with distinct syndromes depending on the hormone secreted. For instance, insulinomas cause Whipple’s triad of hypoglycemia symptoms, while VIPomas and glucagonomas can manifest as diarrhea, diabetes, and necrolytic migratory erythema [15,139]. Unfortunately, many GEP-NENs are non-functional, with no overt hormonal symptoms, and are frequently diagnosed at an advanced stage.

Historically, the diagnosis of GEP-NENs has relied on biomarkers such as chromogranin A (CgA), which is expressed in about 90% of GEP-NETs. While useful, CgA levels can be affected by various factors, and its sensitivity and specificity are far from ideal. As a result, clinicians often depend on additional markers, like synaptophysin, insulin, gastrin, and insulinoma-associated proteins, to refine diagnoses [140,141,142]. Despite this, diagnostic delays remain a global concern. The International SCAN survey by the International Neuroendocrine Cancer Alliance (INCA) highlighted critical knowledge gaps among both clinicians and patients regarding the use of advanced diagnostics, such as ^68^Ga-DOTATATE PET imaging and serum biomarkers, which contribute to late detection and missed opportunities for early intervention [143,144,145]. Compounding this problem are geographic disparities in the availability of key treatments, like PRRT and systemic therapies, which limit access to optimal care in many regions [146].

Recent progress in precision diagnostics offers real promise to address these shortcomings. Blood-based molecular assays, including circulating microRNAs (miRNAs), messenger RNAs (mRNAs), and circulating tumor cells (CTCs), are emerging as sensitive and minimally invasive tools for earlier NET detection and dynamic disease monitoring [147]. Among these, the NETest, an mRNA-based liquid biopsy, has gained significant attention. Compared to CgA, the NETest provides superior accuracy for diagnosing GEP-NENs, assessing residual disease after surgery, and tracking treatment response over time [148]. The latest version of the scoring algorithm, NETest 2.0, has shown improved sensitivity and specificity and has been validated in diverse patient populations worldwide [149]. However, like all diagnostic tools, it is not without limitations; false positives may arise from synchronous gastrointestinal malignancies, and efficacy can vary with tumor grade and stage [150]. The SCAN survey also found that both provider familiarity and infrastructure limitations remain significant barriers to the widespread adoption of novel precision diagnostics.

To provide a clearer synthesis of the concepts discussed earlier, it is helpful to highlight how clinical, pathological, and molecular biomarkers are increasingly guiding treatment choices in GEP-NENs. Poorly differentiated NECs, frequently harbor TP53 and RB1 mutations often along with KRAS or BRAF alteration which are associated with better responses to platinum-based chemotherapy [46,51,151]. In contrast, well-differentiated NETs tend to show mutations in MEN1, ATRX, or DAXX, and are more likely to respond to more targeted therapies such as mTOR inhibitors. While uncommon, cases with high tumor mutational burden or microsatellite instability, especially those with PD-L1 expression may benefit from immune checkpoint blockade [51]. Pathologically, the Ki-67 index remains the cornerstone for grading and treatment guidance, and SSTR expression identifies patients suitable for somatostatin analogs or PRRT [46,152,153]. From a clinical context, tumor site and stage remain equally important, as pancreatic and metastatic tumors require distinctly different approaches compared to early-stage or SI- NENs. Finally, as mentioned earlier, circulating tools like ctDNA and NETest are emerging as valuable assets for tracking resistance and guiding real-time treatment adjustments, reinforcing the move toward precision oncology.

Another layer of complexity in managing GEP-NENs lies in their immune landscape. Most well-differentiated GEP-NETs are considered “cold” tumors, as they lack substantial immune cell infiltration, express low levels of PD-L1, and present few neoantigens [154]. These features help to explain the poor responses observed in clinical trials evaluating immune checkpoint inhibitors such as KEYNOTE-028 and KEYNOTE-158, which reported response rates below 10% for GEP-NENs [155]. While GEP-NENs are largely immune-cold, immune cell infiltration does appear to increase with tumor grade, with higher numbers of PD-1+ T lymphocytes and macrophages noted in more advanced tumors [156].

Interestingly, compared to GEP-NETs, poorly differentiated GEP-NECs often display a contrasting “hot” immune profile. These tumors show greater tumor mutational burden, more robust immune infiltration, and higher PD-L1 expression, all of which can render them more responsive to immunotherapy [157]. Indeed, high-grade NECs, much like melanoma or non-small-cell lung cancer, have shown improved responses to ICIs due to their immunogenic nature and the capacity to reinvigorate exhausted T cells [158]. Unfortunately, this is not the case for most GEP-NETs, which often require combination approaches to reprogram the TME and enhance immunotherapy efficacy. Although data in poorly differentiated GEP-NECs remain limited, a clinical study suggests that combining ICIs with anti-angiogenic agents may help overcome the generally low response rates. For example, in well-differentiated NETs, the trial reported a 15–20% objective response rate with atezolizumab plus bevacizumab, alongside a progression-free survival of 14–15 months [159]. Strategies such as TME modulation, combinatorial regimens, and oncolytic virotherapy are now being actively explored. One notable example is Talimogene laherparepvec (T-VEC), an engineered herpesvirus initially developed for melanoma. When combined with the PD-1 inhibitor, pembrolizumab, T-VEC enhanced CD8+ T-cell infiltration and IFN-γ expression, boosting the effects of PD-1 blockade in clinical studies [160]. This model offers a promising blueprint for overcoming immune resistance in cold GEP-NENs.

**Figure 3:**
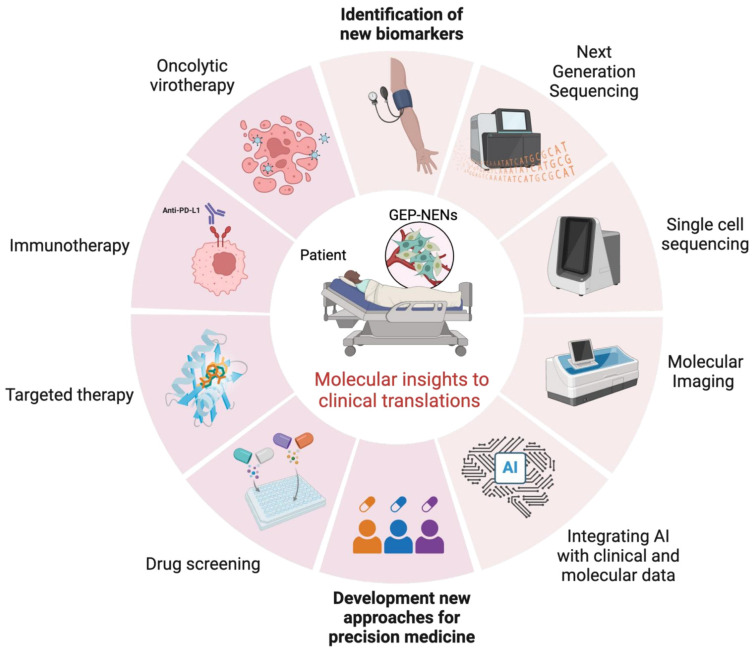
From biomarkers to therapeutics: A roadmap for precision medicine in GEP-NENs.

Figure 3 encapsulates this evolving landscape by illustrating how bridging current clinical practices with emerging technologies can transform the diagnosis and treatment of GEP-NENs. It underscores the shift from conventional, symptom-based management to molecularly informed precision strategies. Technologies like NGS and AI are already proving invaluable in identifying new biomarkers, refining disease classification, and uncovering therapeutic targets. These innovations are expected to facilitate the development of new targeted therapies and more effective immunomodulatory approaches tailored to individual tumor profiles. By embracing such integrative strategies, clinicians can move toward a more personalized model of care, one that not only addresses the biological complexity of GEP-NENs but also improves outcomes across diverse patient populations. Despite these advances, translating AI-driven diagnostics and omics-based stratification into routine clinical practice remains challenging, particularly in low-resource settings. High costs, limited infrastructure, and workforce shortages often hinder implementation, creating disparities in access to precision care. Bridging this gap will require not only continued technological innovation but also strategic investment in training, infrastructure, and global collaboration to ensure equitable adoption across healthcare systems [161,162].

## 6. Conclusions

This review highlights how the convergence of multi-omics, AI, and digital pathology is redefining the landscape of GEP-NEN management. While traditional diagnostic and therapeutic approaches remain essential, they often fall short in capturing the molecular complexity, intratumoral heterogeneity, and dynamic resistance mechanisms characteristic of these tumors. The integration of high-resolution molecular profiling, ML-augmented histopathology, next-generation radionuclide therapies, and rational combination strategies offers a path toward more precise and effective interventions. Several important open questions remain regarding the clinical management and therapeutic strategies for GEP-NENs. Crucially, how can molecular heterogeneity, both within individual tumors and among different tumor sites, be effectively incorporated into grading systems and treatment decisions? How do distinct cellular populations and their spatial distributions within the tumor microenvironment influence clinical outcomes, progression patterns, and resistance to therapy? Advanced molecular omics technologies, including single-cell sequencing, long-read RNA sequencing, spatial transcriptomics, proteomics, and metabolomics, offer powerful approaches to dissecting this complexity. These techniques can precisely characterize tumor subpopulations, identify novel biomarkers, and elucidate cellular interactions within the tumor ecosystem. Integrating these molecular insights into clinical practice will be essential for improving diagnostic accuracy, predicting therapeutic responses, and achieving personalized precision oncology for patients with GEP-NENs.
